# ARL2 is required for homologous recombination repair and colon cancer stem cell survival

**DOI:** 10.1002/2211-5463.13438

**Published:** 2022-05-24

**Authors:** Hani Lee, SeokGyeong Choi, Sojung Ha, Sukjoon Yoon, Woo‐Young Kim

**Affiliations:** ^1^ College of Pharmacy Sookmyung Women's University Seoul Korea; ^2^ Department of Biological Sciences Sookmyung Women's University Seoul Korea; ^3^ Research Institute of Pharmacal Research Sookmyung Women's University Seoul Korea

**Keywords:** ARL2, cancer stem cells, colon cancer, double‐strand repair, homologous recombination repair

## Abstract

ARL2 regulates the dynamics of cytological components and is highly expressed in colon cancer tissues. Here, we report novel roles of ARL2 in the cell nucleus and colon cancer stem cells (CSCs). ARL2 is expressed at relatively low levels in K‐RAS active colon cancer cells, but its expression is induced in CSCs. Depletion of ARL2 results in M phase arrest exclusively in non‐CSC cultured cells; in addition, DNA break stress accumulates in CSCs leading to apoptosis. ARL2 expression is positively associated with the expression of all six RAD51 family genes, which are essential for homologous recombination repair (HRR). Furthermore, ARL2 is required for HRR and detected within chromatin compartments. These results demonstrate the requirement of ARL2 in colon CSC maintenance, which possibly occurs through mediating double‐strand break DNA repair in the nucleus.

## Graphical abstract

A well‐known multifunctional cytosolic protein, ARL2, is also required for homologous recombination repair in the nucleus. The loss of ARL2 selectively eliminated the cancer stem cell (CSC) population. The novel nuclear role of ARL2 may be essential for CSC survival but potentially have less effect on the non‐CSC population. Created with BioRender.com.

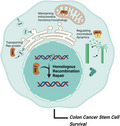

**Abbreviations**
ARL2: ADP ribosylation factor‐like GTPase 2
BCC: bulk‐cultured cells
CSCs: cancer stem cells
DSB: double‐strand break
HRR: homologous recombination repair
NHEJ: nonhomologous end joining
TCGA: The Cancer Genome Atlas

ARL2 (ADP ribosylation factor‐like GTPase 2) is an evolutionarily conserved small GTPase protein that regulates many essential cellular functions. This cargo‐like protein regulates microtubule dynamics, farnesylated/geranylgeranylated G protein translocation to the plasma membrane, and centrosome assembly [1, 2, 3, 4]. In addition, recent investigations have suggested that ARL2 plays an important role in mitochondrial dynamics and fusion [5, 6]. These roles, especially those in RAS activation and centrosome assembly, suggest a positive function of ARL2 in cancer or carcinogenesis. In recent years, interesting roles of ARL2 (both positive and negative) have been identified in cancer [7, 8, 9, 10]. While ARL2 promotes proliferation in breast [7] and colon cancer [8], it suppresses proliferation in glioblastoma [10]. These interesting but contradictory roles of ARL2 observed in different studies are not fully explained by the known cellular function of ARL2, which suggests that there are still some significant biological functions of ARL2 to be identified. The currently known functions of ARL2 are mostly restricted to the cytosol, plasma membrane, and cytosolic compartments, such as mitochondria and ER. Nevertheless, interestingly, nuclear staining of ARL2 has been observed in several previous reports [3, 4], which suggests that ARL2 must perform some function in the nucleus, and this function needs to be determined. The recent finding that STAT3 nuclear retention is mediated by BART/ARL2 may suggest that ARL2 exerts one of its effects in the nucleus [11] of immune cells.

A few small GTPase and the regulators play both oncogenic and tumor‐suppressive roles depending on the cellular context [12, 13]. Germline mutation of a DNA repair gene, BRCA1/2, predisposes individuals to the development of ovarian and breast cancers by inducing genomic instability and enhances the susceptibility of cancer to treatment with PARP inhibitors [14]. Therefore, genes related to DNA repair may contribute to cancer, thus acting as a double‐edged sword [15, 16].

Not all cells in a tumor have an equivalent cancer formation capacity *in vivo*. Indeed, the fact that only a very small proportion of cells can generate tumors *in vivo* is the basis of the concept that so‐called ‘cancer stem cells (CSC)’ develop and differentiate into ‘cancer tissue’. Virtually, all human malignant tumors, including nonsolid tumors, have a CSC population that is stimulated by most chemo‐ and radiotherapies; therefore, these cells are believed to be responsible for resistance, recurrence, and even metastasis. Over the past decade, numerous investigations have been performed to achieve the selective and/or effective targeting of CSC. However, very few of these studies were successful in proceeding to the next level of drug development, and limited candidates are currently being clinically evaluated. Recent findings suggest that the many characteristics of CSC, mesenchymal cells from cancer epithelial‐to‐mesenchymal transition, and dormant or slow‐growing cells may overlap, and indeed, some of these cells are interchangeable in actual tumors, especially during the process of acquiring resistance to therapies [17]. However, it is still widely accepted that CSC shares many characteristics of their own tissue (adult) stem or progenitor cells [18, 19].

We previously screened CSC‐specific vulnerable targets using 5000 targetable siRNAs and found that five genes are essential for the growth of glioblastoma multiform CSC [20]. One of these genes was ARL2, which is preferentially required in glioblastoma cell line‐derived CSC compared with conventional bulk‐cultured cells (BCC: cultured in medium supplemented with 10% FBS, surface‐attached, without enrichment of CSCs) of the same cell line. Because we can postulate that known microtubule‐ and centrosome‐related functions are common in CSC and BCC, these results suggest the possibility that this cargo protein plays an unknown but essential role in CSC that is dispensable in BCC.

In the present study, we investigated an unknown role of ARL2 in the nucleus, especially in the nucleus of human colon cancer stem cells. Through the analysis of public databases of human tissues, we demonstrated that ARL2 may play an important role in human colon cancer tissues and normal tissues and that ARL2 is related to stem cell properties and performs previously unknown nuclear functions. Using CSC enrichment culture and biochemical analysis, we demonstrated that ARL2 is more strongly required by CSC than by BCC and that ARL2 is involved in homologous recombination repair (HRR). Collectively, this research is the first in‐depth demonstration of the role of ARL2 in the nucleus, which is required for HRR, and this role is more essential for CSC maintenance than for that of non‐CSC compartments in tumors. This study revealed a novel and important function of ARL2 and suggested that blockade of the HRR process may more strongly affect the CSC population.

## Materials and methods

Information about the antibodies and reagents used in this study is listed in Table S1.

### Cell culture

The human colon cancer cell lines KM12, HT29, HCT116, SW480, and HCT15 were obtained from the National Cancer Institute (NCI, Frederick, MD, USA). The fibrosarcoma cell line HT1080 was purchased from Korean Cell Line Bank (KCLB, Seoul, Korea), and HEK293T was obtained from American Type Culture Collection (ATCC, Manassas, VA, USA). TRI‐DR U2OS was a gift from Dr. Oberdoerffer [21]. The cells were cultured in DMEM or RPMI 1640 supplemented with 10% FBS (Thermo Fisher Scientific, Waltham, MA, USA) in cell culture‐treated flasks for maintenance and for BCC culture. CSC was enriched in DMEM/F‐12 (Thermo Fisher Scientific) supplemented with EGF, basic fibroblast growth factor, and B27 supplement on poly HEMA (polymer of 2‐hydroxyethyl methacrylate)‐treated culture dishes as previously described [22]. Both media were supplemented with 1% penicillin and streptomycin (Welgene, Gyeongsan, Korea).

### RNAi transfection

RNAi molecules that effectively reduced only target protein expression were identified by western blotting. Transient siRNA transfections were carried out with TransIT‐X2 (Mirus Bio LLC, Madison, WI, USA) or RNAiMAX (Thermo Fisher Scientific), and analyses were performed 3–5 days after siRNA transfection. Nontargeting control siRNA (siNC, Cat# 51‐01‐14‐04, 5′‐CGUUA AUCGC GUAUAAU ACGCGU‐3′) and siRNA targeting ARL2 were obtained from Integrated DNA Technologies (IDT, Coralville, IA, USA). The following siRNAs were used in this study: siARL2 #1 (Cat# hs.Ri.ARL2.13.1, 5′‐AUCCU UCACU CAGUU GU‐3′), siARL2 #2 (Cat# hs.Ri.ARL2.13.2, 5′‐GGUCC CUCAC CUUCA CC‐3′), and siBRCA2 (Cat# hs.Ri.BRCA2.13.1, 5′‐CAAGA AGCAU GUCAU GGUAA UACTT‐3′). The following lentiviral shRNA plasmids were purchased from Sigma Aldrich (Burlington, MA, USA): shNC (Cat# SHC001); shARL2 #1 (Cat# TRCN0000048026); and shARL2#2 (Cat# TRCN0000286711). shRNA expressing lentiviruses were produced in HEK293T cells by co‐transfection of the shRNA expressing vector with pMD2.G (Addgene, Watertown, MA, USA, #12259) and psPAX2 (Addgene #12260) using jetPrime (Polyplus, Illkirch, France), and transduced into a colon cancer cell line with 5 μg·mL^−1^ hexadimethrine bromide.

### Western blotting

For western blotting, cellular proteins were extracted with T‐PER™ Tissue Protein Extraction Reagent (Thermo Fisher Scientific) supplemented with a proteinase inhibitor cocktail, cOmplete™ (Millipore Sigma, Burlington, MA, USA), and the phosphatase Inhibitor. The proteins were separated by SDS‐polyacrylamide gels and transferred to PVDF membranes (Millipore Sigma). The membranes were blocked with 5% nonfat milk and then incubated with primary antibodies overnight at 4 °C in Tris‐buffered saline supplemented with 5% of bovine serum albumin and 0.04% Tween 20. After washing the membranes with the same buffer, the membranes were incubated with HRP‐conjugated secondary antibodies for 1 h at RT and developed with ECL reagents. The bands were visualized on X‐ray films or an Amersham Imager 680 (GE Lifescience, Chicago, IL, USA).

### HRR reporter, cell cycle, and apoptosis assay

The HRR reporter assay was performed in the doxycycline‐inducible TRI‐DR U2OS cell line. The cells were treated with/without 10 μg·mL^−1^ doxycycline after siRNA transfection. The doxycycline‐induced translocation of I‐Sce1 into the nucleus increased the number of dsDNA breaks, and only HRR‐mediated DNA repair could generate GFP‐positive cells [23]. The HRR efficiencies were analyzed by flow cytometry (FACSCalibur, BD, San Jose, CA, USA) after 48 h of treatment by assessing the fraction of GFP‐positive cells. Data analysis was performed using flowjo (FlowJo LLC, Ashland, OR, USA). graphpad prism v9 (GraphPad Software, San Diego, CA, USA) was used for statistical analysis. Multiple comparisons were performed with a one‐way ANOVA, and the graph presents the data as the mean ± SEM. Cell cycle and apoptosis assay were performed as previously reported [24].

### Subcellular fractionation

Fractionation was performed as previously described [25, 26] with slight modification. Briefly, cells were resuspended and disrupted in low‐salt buffer A (10 mm HEPES [pH 7.9], 10 mm KCl, 0.1 mm EDTA, 0.3% NP‐40, and protease inhibitor cocktail on ice for 5 min, and the nuclei were collected by centrifugation (5 min, 800 ***g***, 4 °C). The supernatants were collected as the cytosolic extract (CE). The nuclei were washed three times in buffer A without NP‐40, and the proteins were extracted for 10 min on ice in high salt buffer B (20 mm HEPES [pH 7.9], 0.4 m NaCl, 1 mm EDTA, 25% glycerol, and protease inhibitor cocktail) after sonication. Nuclear high salt‐soluble and high salt‐insoluble extracts were separated by centrifugation (5 min, 14 000 ***g***, 4 °C). The supernatants were collected as the nuclear high salt‐soluble extracts, which included most extracted histones (Nuclear soluble extract, NE sol). The insoluble fractions containing insoluble chromatin were washed three times in buffer B and denatured by incubation in the SDS sample buffer (1% SDS, 20 mm Tris [pH 7.9], 0.15 m NaCl, 1 mm EDTA, 10% glycerol, 15 min, 75 °C). Then, the insoluble nuclear extracts were further clarified by centrifugation (15 min, 16 000 ***g***, 4 °C). The supernatants were collected as the nuclear insoluble extract (NE insol). The yet‐insoluble nuclear matrix pellets that require stronger denaturing agents such as 10 m urea, to be dissolved were not analyzed in this fractionation experiment [27].

### Bioinformatics analysis

Data on protein expression levels in patients were collected from the Human Protein Atlas (available from www.proteinatlas.org; accessed on November 2021) [28]. For each cancer type, the patients with ‘high’ or ‘medium’ ARL2 protein expression levels as determined by immunohistochemistry (validated antibody: HPA044610) were considered to be positive for ARL2 protein expression. ARL2 mRNA expression in various cancer types, KRAS mutation status, and mRNA expression level were assessed with data from TCGA PanCancer Atlas. The cBio Cancer Genomics Portal was used to establish correlations with patient K‐RAS activity. q‐omics software (http://qomics.sookmyung.ac.kr) [29] was used to analyze normal colon tissues and colon cancer tissues or to compare gene expression levels according to the ARL2 expression level.

## Results

**Fig. 1:**
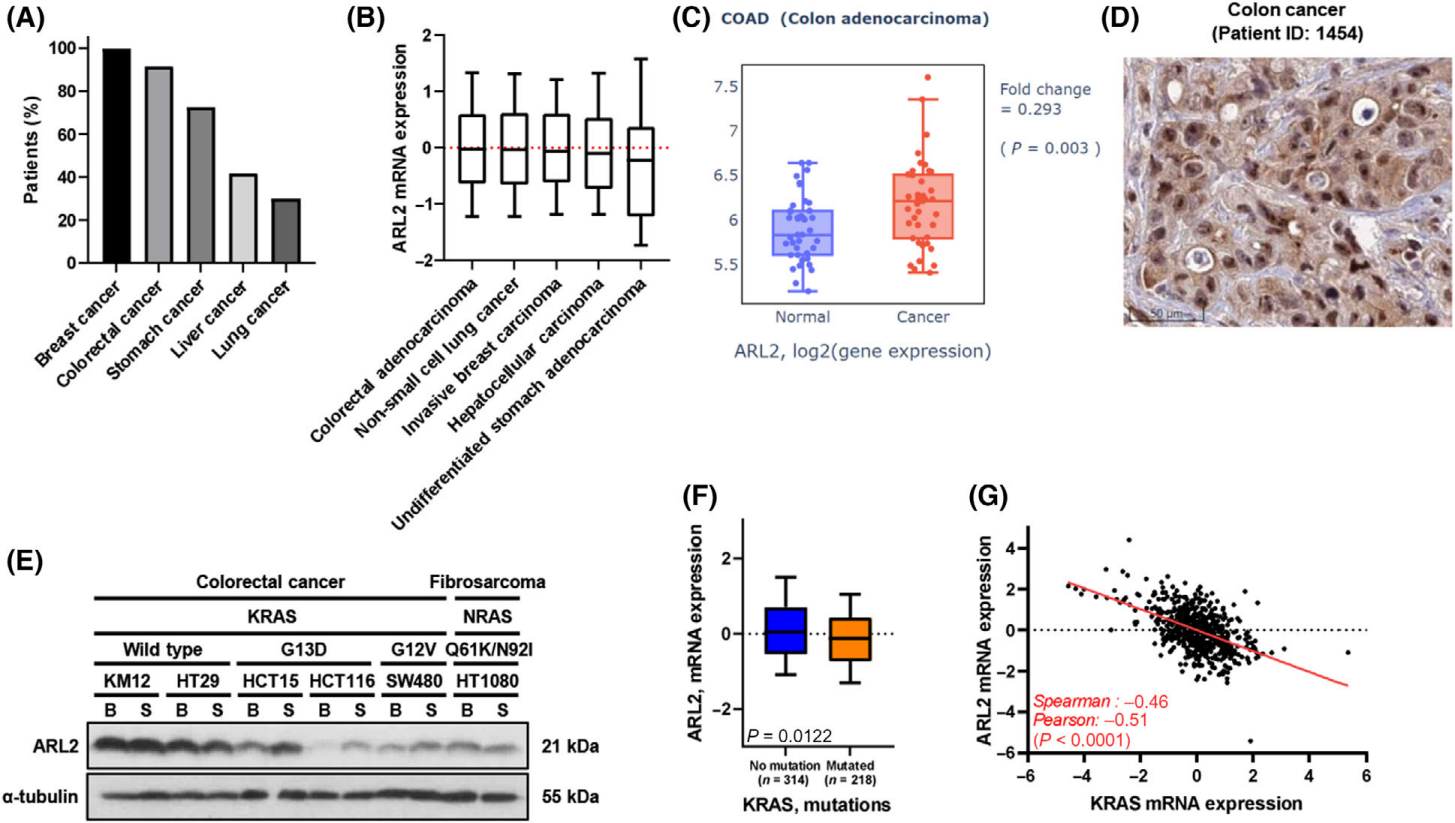
ARL2 is highly expressed in the nucleus of colon cancer tissues. (A) Analysis of ARL2 protein expression among five highest mortality cancers. The graph displays the percentage of patients being positive for ARL2 by IHC (validated antibody: HPA044610) for each cancer type. The data are from the Huma Protein Atlas [28]. (B) The ARL2 mRNA expression in various cancer types. Boxes show the median and interquartile, while bottom and top bars of the whisker indicate the 10–90 percentile range. The mRNA expression *z*‐score relative to all sample (log RNA seq V2 RSEM) in TCGA PanCancer Atlas was analyzed. (C) The mRNA expression of ARL2 in the normal and cancer colon adenocarcinoma sample in TCGA (*n* = 39). Data were retrieved from q‐omics. The *P*‐value from Student's *t*‐test is indicated. (D) Example of nuclear staining seen in a colon cancer specimen (https://www.proteinatlas.org/ENSG00000213465‐ARL2/pathology/colorectal+cancer#imid_12184721). Scale bars, 50 μm. (E) Expression of ARL2 dependent on RAS mutation status in cells in different population. B, bulk‐cultured cells; S, CSC spheres. (F) The ARL2 expression level in KRAS WT tumors (*n* = 314) *versus* tumors with KRAS mutant (*n* = 218) analyzed from the data from TCGA. Boxes show the median with interquartile, and bars indicate 10–90 percentile range. Unpaired *t*‐test *P*‐value is indicated. (G) Correlation between the mRNA expression *z*‐scores relative to all samples (log RNA Seq V2 RSEM) of ARL2 and KRAS. The data are from TCGA. Dots in graph represent individual tumors (*n* = 592). [Colour figure can be viewed at wileyonlinelibrary.com]

To investigate the role of ARL2 in cancer and cancer stem cells, we attempted to select the most relevant cancer tissues based on clinical data available in public databases. Currently, the five cancers that are associated with the highest mortality rates worldwide are breast‐, colorectal‐, stomach‐, lung‐ and liver‐derived cancers [30]. Therefore, we compared the immunohistochemical staining results of human cancer tissues available in the Human Protein Atlas (http://www.proteinatlas.org). The results showed that breast and colon cancer exhibited stronger staining than cancers derived from other tissues (Fig. 1A and Fig. S1). mRNA expression data extracted from TCGA (https://www.cancer.gov/tcga) were also analyzed. Among the five most fatal cancers, colon cancer expressed the highest levels of ARL2 mRNA (Fig. 1B). ARL2 mRNA expression was also significantly higher in colon cancer tissues than in adjacent normal tissues (Fig. 1C). Based on these findings, we concluded that ARL2 must play an important role in colon cancer. Interestingly, we found that in both normal tissues (https://www.proteinatlas.org/ENSG00000213465‐ARL2/tissue/colon) and cancer tissues (Fig. 1D), ARL2 was predominantly localized in the nucleoplasm. In the cell line staining results in the Human Protein Atlas (https://www.proteinatlas.org/ENSG00000213465‐ARL2/subcellular), cell lines derived from other tissue also expressed ARL2 in the nucleus.

Human normal colon tissue single‐cell RNA‐seq data were examined (Fig. S2). One enterocyte group (out of 7) and one undifferentiated cell group (out of 3) expressed high levels of ARL2 and showed a largely overlapping gene expression pattern (especially in terms of the low expression of differentiated enterocyte markers and high expression of undifferentiated marker genes) (Fig. S2). These findings suggest that two populations in the normal colon at the early differentiation or undifferentiated stages express high levels of ARL2. Therefore, we next examined the expression of ARL2 in several cancer cell lines under bulk‐cultured cells (BCC) conditions or CSC‐enriched cultured conditions. The induction of ARL2 expression in CSC was observed in a subset of colon cancers, while basal ARL2 expression in BCC was low (Fig. 1E). Surprisingly, the cells that expressed low levels of ARL2 but presented an increase in CSC were all K‐RAS mutant cells, which harbor G13D or G12V activating mutations. The NRAS mutated cell did not show this pattern. Since ARL2 facilitates RAS activation, we next examined whether an inverse association between K‐RAS activation mutation and ARL2 expression is observed in human colon cancer tissues. Similar to the cell line data, the human colon cancer tissue data also showed that the tumors harboring the K‐RAS‐activating mutation expressed significantly lower levels of ARL2 (Fig. 1F). Surprisingly, we also found that the expression of these two genes showed a strong inverse correlation (Pearson correlation coefficient, −0.51; *P* < 0.001) in human colon cancer tissues (Fig. 1G). Therefore, in colon cancer cells with higher K‐RAS activity (with higher expression or activation mutation), ARL2 expression may be increased in CSC.

**Fig. 2:**
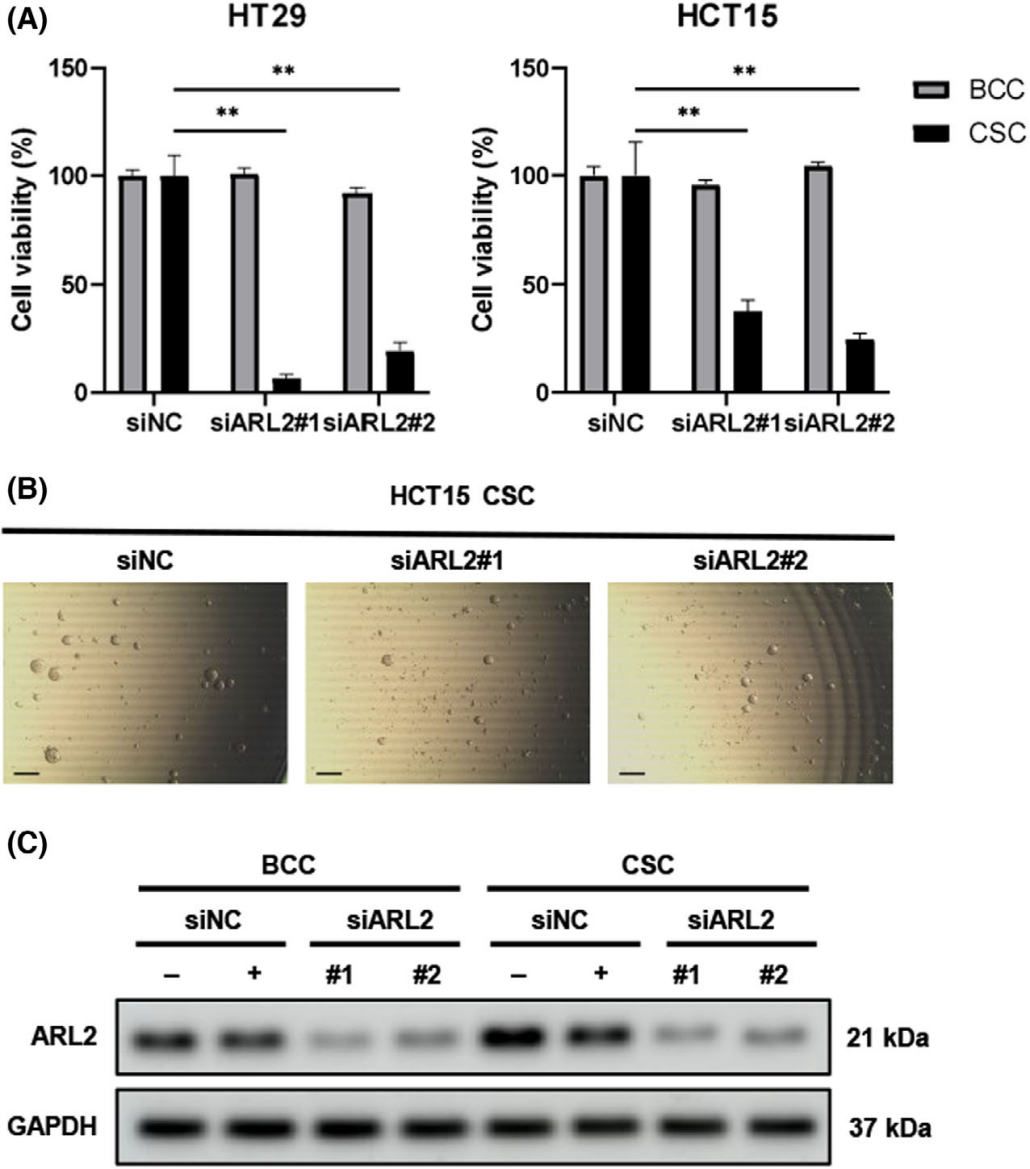
ARL2 is required for colon cancer CSC survival. (A) siRNAs targets ARL2 were transfected to colon adenocarcinomas cell lines and divided to BCC (bulk‐cultured cells, attached with FBS) or CSC (cancer stem cells, nonattached stem cell media) conditions. After 3 and 5 days, respectively, the number of cells or spheres (over 100 μm in diameter) were counted. The data represent mean ± SEM, Student's *t*‐test; *n* = 6; **P < 0.01. (B) The representative images of CSC sphere are shown. Scale bars, 200 μm. (C) The efficiency of siRNA in ARL2 *vs* negative control (NC) expression is shown. NC, nontargeting negative control. [Colour figure can be viewed at wileyonlinelibrary.com]

Since our previous finding from a screen of glioblastoma [20] and the data in Fig. 1 of this study suggested an important role of ARL2 in the progenitor/stem‐like population of normal colon tissues and colon cancer tissues, we next directly examined the impact of silencing ARL2 expression in BCC and CSC using the colon cancer cell lines HCT15 and HT29, which harbor mutant and wild‐type K‐RAS, respectively. While the survival of BCC cells was not considerably suppressed, the CSC colonies almost completely disappeared after transfection with siRNA targeting ARL2 (Fig. 2A,B). Both ARL2 siRNAs effectively suppressed ARL2 protein levels (Fig. 2C).

**Fig. 3:**
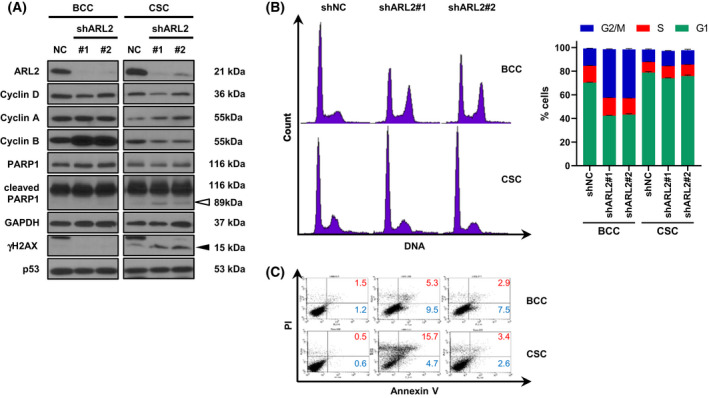
ARL2 is required for colon CSC spheres survival and genome stability. shRNA expressing lentivirus were purified and infected to colon adenocarcinoma HT29 and cultured as BCC (bulk‐cultured cells) or CSC (cancer stem cells) conditions. (A) Western blotting showed that both ARL2 shRNAs efficiently increased cyclin B in BCC only. An apoptosis marker cleaved PARP1 was only found in CSC. The marker of DNA damage, γH2AX is only identified in CSC and further increased by both ARL2 shRNAs. Open and closed arrowheads represent cleaved PARP‐1 and γH2AX, respectively. (B) The ARL2 shRNAs arrested cell cycle at G2/M in BCC but did not in CSC. Left, representative histogram. Right, quantitative graph. (C) The ARL2 shRNAs did not induce cell death yet in BCC, while it increased cell apoptosis in CSC. The early (blue) and late (red) apoptotic cell percentages are indicated. NC, nontargeting negative control. [Colour figure can be viewed at wileyonlinelibrary.com]

We then investigated the mechanism underlying the differences between BCC and CSC after silencing of ARL2 expression. We used a lentivirus carrying a new sequence of ARL2 shRNA to avoid transfection‐related toxicity. We observed cyclin B accumulation only in the ARL2 shRNA virus‐infected BCC, and no other cyclins were significantly affected by ARL2 shRNA. The accumulation of cyclin B (M cyclin), but not the accumulation of cyclin A (G2 cyclin), and the blockade of the cell cycle in the G2/M phase were consistent with the previous finding that ARL2 is required for centrosome placement [3, 4]. Most surprisingly, neither accumulation of cyclin B nor changes in cell cycle progression were observed in CSC. However, we observed that apoptotic signatures (PARP cleavage and Annexin V staining) were induced by ARL2 shRNA only in CSC. In the western blotting analysis, we obtained an unexpected result that the level of phosphorylated H2AX (γH2AX, a surrogate marker of DNA double‐strand breaks [DSBs]) was increased by ARL2 shRNAs (Fig. 3). Interestingly, the basal γH2AX levels were higher in the CSC, as reported in embryonic and pluripotent stem cells [31, 32].

**Fig. 4:**
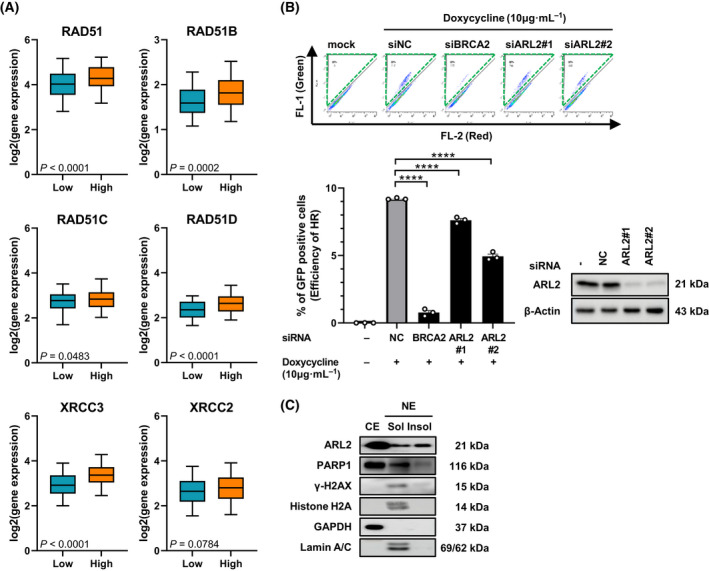
ARL2 is associated and required for the HRR process in nucleus. (A) Box plots of ARL2 log2 gene expression *vs*. 6 RAD51 family genes are retrieved among colon adenocarcinoma case (*n* = 430) by using the q‐omics. The data were from TCGA. Boxes show the median and interquartile with 5–95 percentile range. Unpaired *t*‐test *P*‐values are indicated. (B) HRR reporter assay was carried out in the doxycycline‐inducible TRI‐DR U2OS cells. The cells were transfected with indicated siRNA, and the percentage of GFP‐positive cells were analyzed using flow cytometry. Two‐color fluorescence analysis was performed to quantify the green fluorescent cells indicated with green dotted box (top). The quantitative graph represents mean ± SEM, one‐way ANOVA; *n* = 3; ****P < 0.0001 (lower left). Western blot analysis shows ARL2 expression in TRI‐DR U2OS following siRNA knockdown (lower right). (C) HT29 proteins were fractionated into cytoplasmic, nuclear high salt‐soluble, and insoluble fractions. CE, cytosolic extract; Insol, insoluble; NC, non‐targeting negative control; NE, nuclear extract; Sol, soluble. [Colour figure can be viewed at wileyonlinelibrary.com]

DNA DSBs have a serious negative impact on the integrity of the genome in cells. Therefore, cells try to fix these breaks as soon as possible using NHEJ (nonhomologous end joining) or HRR (homologous recombination repair). The increase in the γH2AX levels in the CSC after the knockdown of ARL2 expression may indicate that some DNA DSBs are not successfully repaired in these CSC. To investigate the potential role of ARL2 in DNA DSB repair, we examined whether any of the DNA DSB repair machineries are associated with ARL2 expression in colon cancer tissues. Of the many DSB repair‐related genes we investigated, the association between the expression of ARL2 and RAD51 family genes was most evident. Among the six somatic RAD51 family genes in humans, the expression of five genes showed a significant positive association with the expression of the ARL2 gene (Fig. 4A). This correlation did not reach statistical significance for only one gene, XRCC2 (*P* = 0.078). RAD51 and all five paralogs together play important roles as complexes in single‐stranded regions during HRR and replication [33]. Therefore, this result suggests that the ARL2 protein may be related to RAD51 functions in HRR. We used a widely used and quantitative reporter system to assess HRR efficiency after RNAi administration. The doxycycline‐inducible model cell line, TRI‐DR U2OS [21], was used for the HRR efficiency test, and after being subjected to ARL2 knockdown, these cells showed a significant decrease in the proportion of GFP‐positive cells, which indicates the efficiency of the HRR process (Fig. 4B). Finally, we tested whether ARL2 is indeed present in the chromatin structure in the nucleus. The known chromatin‐associated proteins lamin A/C [34, 35, 36] and histone H2A were detected in the high salt‐soluble nuclear fractions. ARL2, PARP1, and γH2AX were also detected in the high salt‐insoluble nuclear fractions that contain chromatin components, including many repair proteins (Fig. 4C) [25, 26, 37, 38]. Therefore, we conclude that ARL2 is required for the HRR process and that functional relationships between RAD51 family genes may exist in the nuclear chromatin environment.

## Discussion

We analyzed public databases and observed the nuclear expression of ARL2 in colon cancer tissues. In addition, the negative association between ARL2 expression and K‐RAS activity was demonstrated with its CSC‐specific roles. The requirement of ARL2 in the CSC of colon cancer was shown in two cancer cell lines, revealing that ARL2 knockdown‐induced genomic DNA break‐related stress and apoptosis are limited to the CSC population, while ARL2 knockdown arrests the cell cycle of BCC in the M phase, as previously suggested based on the known cytosolic effect of ARL2 on microtubules/centrosomes. CSC‐specific DNA break‐related stress was caused by the absence of ARL2‐associated DSB repair. The strong positive association between most RAD51 family genes in human cancers indicates the potential role of ARL2 in HRR. Finally, the physical presence of the ARL2 protein with other chromatin proteins in the soluble and insoluble nuclear fractions indicates that this protein may contribute to the DNA DSB repair process in chromatin.

In recent decades, the progression of knowledge about adult stem cells has revealed many important characteristics of stem or progenitor cells in normal tissue development and regeneration. Many cancers appear to develop from this population of cells, and some of these cells differentiate further in tumors while recapitulating the differentiation of the tissue of origin. While these further differentiated cancer cells divide quickly and are targets of conventional cancer therapies, undifferentiated stem cell‐mimicking cells, CSC, behave as if they are normal tissue stem cells [18, 19]. These cells grow slowly and secrete many xenobiotics, leading to the evasion of conventional cancer therapies. Therefore, CSC should be eliminated in order to achieve a complete cure and prevent the development of resistant recurrence. In attempts to identify novel targets for new CSC‐specific therapy, we previously identified ARL2 as a candidate gene for targeting glioblastoma CSC [20]. In the current study, we presented evidence that ARL2 can be a potential target for targeting CSC in the treatment of colon cancer.

The subcellular localization of specific proteins indicates their main functions. This is because the subcellular localization of a protein is critical for it to perform its biological function. For example, many germline mutations of CFTR that disrupt the channel protein's plasma membrane localization cause severe disease [39]. Although the nuclear localization of ARL2 was previously described, its role in the nucleus was not well addressed before this report. Our finding that ARL2 resides in the nucleus of human tissues and in the insoluble nuclear/chromatin fraction proved that this protein might play important role in the nucleus. However, our fractionation results from the cell lines also indicate that many ARL2 proteins remain in the cytosol, probably playing known cytosolic roles. In addition, it may suggest some differences between the cells in ‘*In vitro’* and ‘*In vivo*’ conditions: for example, in cell growth and microenvironment. Here, we demonstrated that ARL2 is required for HRR, which is an exclusive nuclear event. The unique positive association between the expression of ARL2 and that of most RAD51 family genes is interesting and strongly suggests that it plays a positive role in HRR. All of the RAD51 family proteins mediate strand displacement in the HRR as complexes [33] and play an important role in the replication fork [40], similar to many other HRR proteins. In the current report, only an association between the expression of ARL2 and that of RAD51 family members was shown. The detailed molecular mechanism by which ARL2 is involved in the HRR needs to be addressed.

It is somewhat surprising that ARL2 knockdown did not affect the cell cycle of CSC, although it induced apoptosis in CSC. ARL2 knockdown arrests BCC cells in the M phase and increases cyclin B levels [6]. However, neither a change in cell cycle progression nor the accumulation of cyclin B was observed in CSC, which suggests two possibilities. First, centrosome localization is not mediated by ARL2 in CSC. Second, cells can die before they reach the M phase. The HRR mechanism may be the same in CSC and BCC. HRR requires the correct template DNA and therefore can only be mediated during the S and G2 phases, while NHEJ can be mediated throughout the cell cycle and mainly occurs in the G1/G0 phase. The failure of DNA DSB repair by HRR in the absence of ARL2 should be common in both BCC and CSC. However, only CSC exhibited the accumulation of γH2AX and apoptosis. If CSC experiencing problems with HRR die quickly at the S/G2 phase, they may not reach and accumulate in the M phase. However, if BCC cells avoid these problems by employing other DSB repair mechanisms, such as NHEJ, they can pass to the next stage, the M stage, and face another problem in centrosome misallocation. Based on these findings, we suggest that CSC are more vulnerable to HRR failure than BCC. While HRR maintains genomic integrity, error‐prone NHEJ dominantly mediates DNA DSB repair in most cells in general. The germline mutation frequencies are lower than those in somatic cells *in vivo* [41]. The maintenance of genome integrity is essential for the self‐renewal of embryonic stem cells [42]. In that regard, it is likely that stem cells may prefer HRR [43] over NHEJ to avoid undesirable mutations. Indeed, embryonic stem cells express many more HRR genes [44, 45] and fewer NHEJ genes than differentiated cells. Stem cells experience a longer S phase and a slower cell cycle than differentiating cells in order to facilitate HRR [44, 45]. Therefore, the preference for HRR over NHEJ in embryonic and pluripotent stem cells has already been suggested [43, 45]. One report showed that breast CSC may prefer HRR due to cell cycle distribution [46]. Another report showed that RAD51 knockdown synergizes with the effects of chemotherapy on gastric cancer, suggesting the importance of HRR in CSC [47]. However, the DNA DSB repair preference of CSC is not well understood. As previously mentioned, CSC shares many characteristics with normal stem cells. Current findings suggest that the HRR may be exclusively preferred by CSC, which is why CSC critically depends on the HRR. This may be because ever‐growing stem cells would die rather than accumulate mutations, which is different from life‐limited differentiating cells.

Our finding of the negative correlation between K‐RAS activity and ARL2 expression is interesting. The oncogenic gain‐of‐function mutation of K‐RAS may be sufficient to maintain high activity with low ARL2 expression. The presence of this oncogenic mutation was considered to be a surrogate marker of K‐RAS activity. However, recent findings in breast and lung cancers suggest that K‐RAS gene expression also represents its activity [48, 49]. BCC with active K‐RAS had low ARL2 expression, but ARL2 expression was increased in CSC, which suggests that higher ARL2 expression is necessary for CSC. This may be related to K‐RAS activity because K‐RAS is a pivotal player in the CSC of many cancers [50, 51, 52, 53, 54]. K‐RAS drives radioresistance via enhanced HRR and NHEJ due to NRF2 activation and subsequent RAD51 and 53BP1 transcription [55, 56]. Accordingly, K‐RAS‐mutant colorectal cancer cells are highly dependent on RAD51 and HRR for survival [57]. These results suggest a requirement for ARL2, possibly through the transportation of activated RAS family members. However, our identification of the ARL2 protein in both the high salt‐insoluble and high salt‐soluble nuclear/chromatin fractions suggests that ARL2 plays additional role in the nucleus. The unexpected role of activated H‐RAS in the nucleus, which requires prenylation and regulates cell cycle progression, suggests that these two proteins can work together in the nucleus and can be used to affect K‐RAS. Further investigation using mutant ARL2 that cannot localize to the nucleus and examination of HRR efficiency may be warranted.

## Conclusions

Our investigation presented herein supports two novel hypotheses for the first time. First, ARL2 is a new HRR mediator of nuclear proteins. Second, the HRR is more critical and essential for CSC survival than for the survival of other tumor cells. Our findings strongly support the possibility that ARL2 and HRR can be new therapeutic targets specific for colon CSC; additionally, these findings indicate the urgent need for further investigation of the molecular mechanisms and clinical applications of ARL2 in HRR.

## Conflict of interest

The authors declare no conflict of interest.

## Author contributions

W‐YK and SY conceptualized and supervised the study; W‐YK, SY, HL, and SC designed the experiments; HL, SC, and SH performed the experiments; W‐YK, HL, and SC wrote the manuscript. W‐YK, SC, and SH revised the manuscript.

## Supporting information


**Fig. S1.** The relative expression of ARL2 in human cancer specimen.
**Fig. S2.** Expression ARL2 in human colon cells.
**Table S1.** List of antibodies and reagents used in this study.

## Data Availability

The data that support the findings of this study are available from the corresponding author (wykim@sookmyung.ac.kr) upon reasonable request.
