# Supplementary material for: ARL2 is required for homologous recombination repair and colon cancer stem cell survival
Source: FEBS Open Bio. 2022 May 24;12(8):1523–33. doi: 10.1002/2211-5463.13438 (PMC9340879; doi:10.1002/2211-5463.13438)
Supplement: Supplementary file 1 — Fig. S1. The relative expression of ARL2 in human cancer specimen. Fig. S2. Expression ARL2 in human colon cells. Table S1. List of antibodies and reagents used in this study. [file FEB4-12-1523-s001.docx]

**SUPPLEMENTARY MATERIALS**

List of Supplementary Materials.

- Figure S1 and legend
- Figure S2 and legend
- Table S1 and title
- References of Supplementary Materials

**Figure S1.** The relative expression of ARL2 in human cancer specimen

The representative IHC images of ARL2 in each cancers are obtained from the Human Protein Atlas dataset (<https://www.proteinatlas.org/ENSG00000213465-ARL2/pathology>). Scale bars, 200μm.


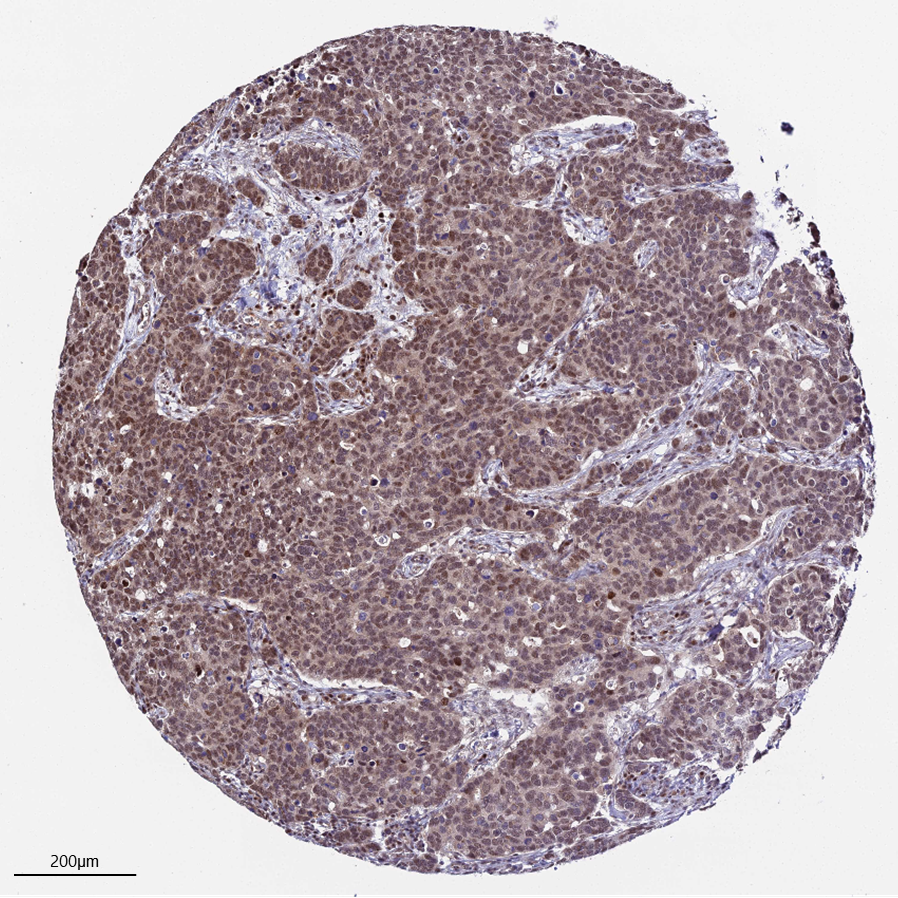

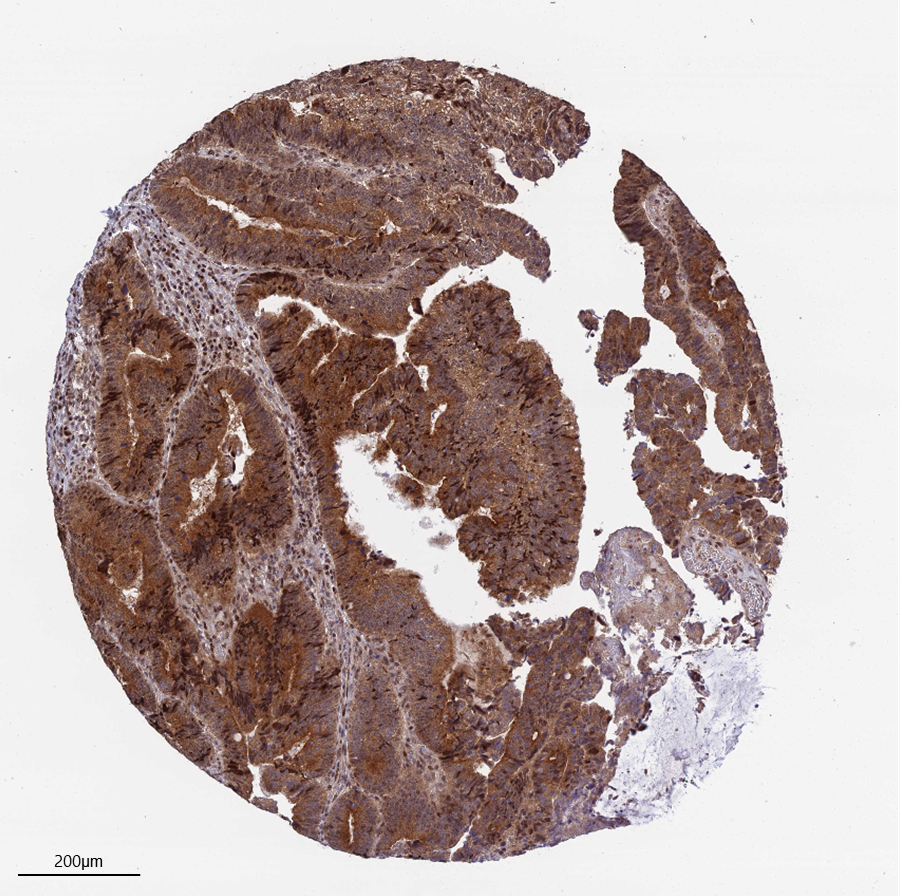

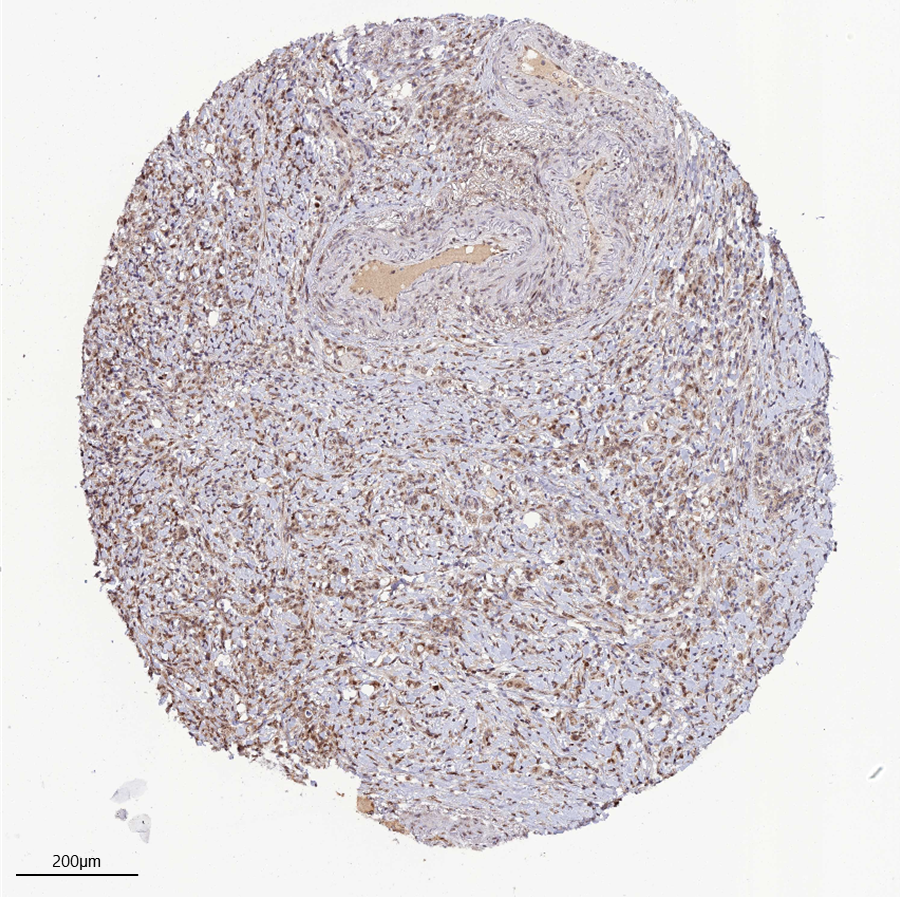

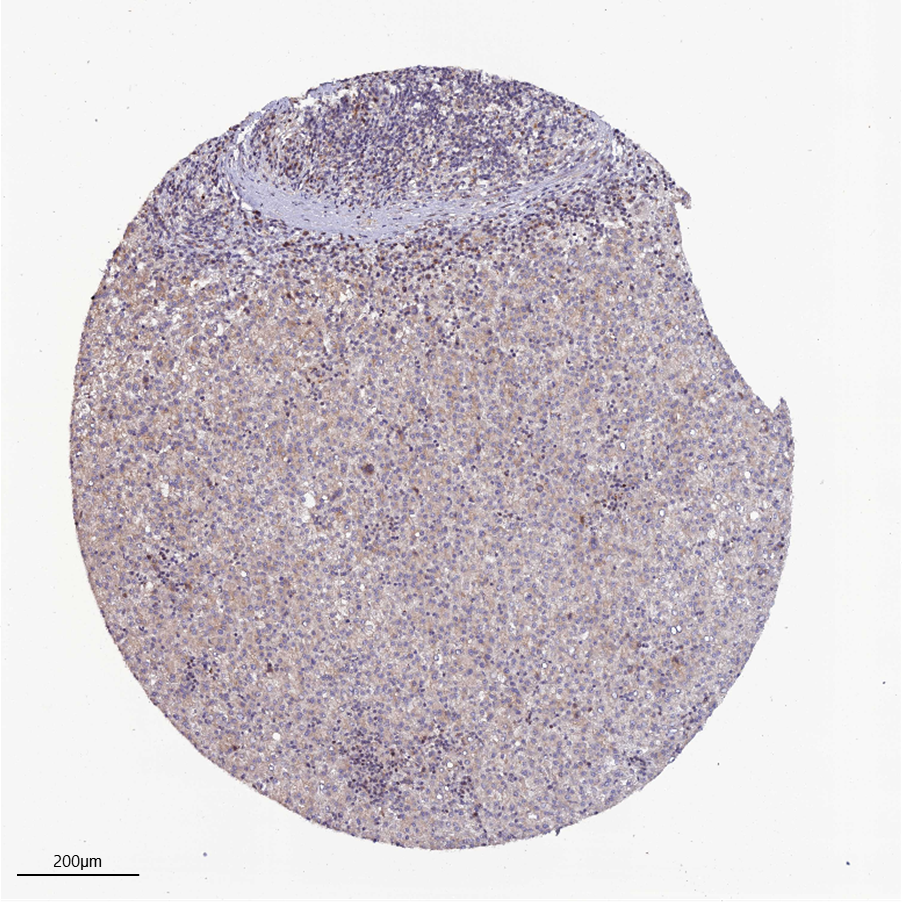

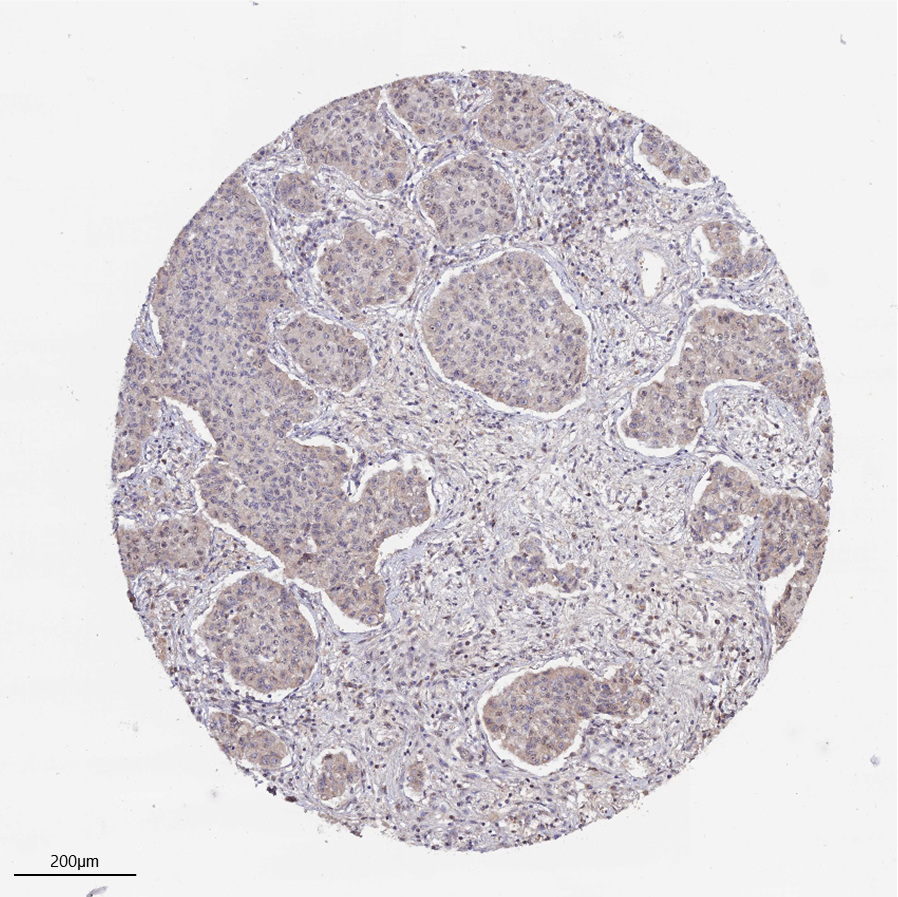


**Breast Cancer**

**(Patient ID: 2160)**

**Colon Cancer**

**(Patient ID: 97)**

**Stomach Cancer**

**(Patient ID: 2626)**

**Liver Cancer**

**(Patient ID: 2177)**

**Lung Cancer**

**(Patient ID: 2779)**

**Figure S2.** Expression ARL2 in human colon cells

Single cell transcriptomic data that include healthy human tissue for the cell type specificity was obtained from Human Protein Atlas [[1](#_ENREF_1)]. **A)** RNA expression in the single cell type clusters identified in colon tissue [[2](#_ENREF_2)] visualized by a UMAP plot. UMAP plot visualizes the cells in each cluster; where each dot corresponds to a cell with a unique color to each cluster and intensity, which color the individual cells according to % of max. **B)** The heatmap shows expression of ARL2 (on top) and well-known cell type markers. The fraction of highest expression is used in the heatmap analysis. The data accession and download was performed November 2021. (<https://www.proteinatlas.org/ENSG00000213465-ARL2/single+cell+type/colon>).

**
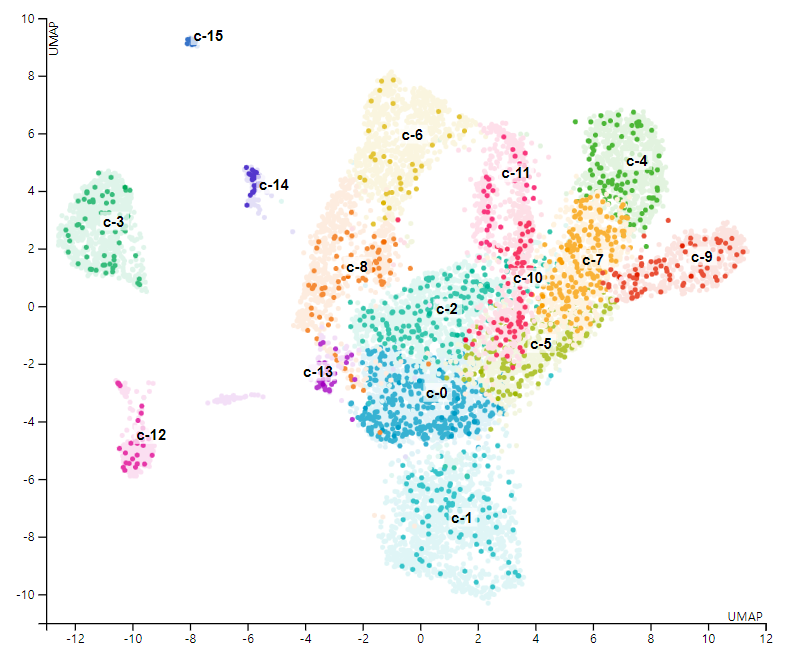

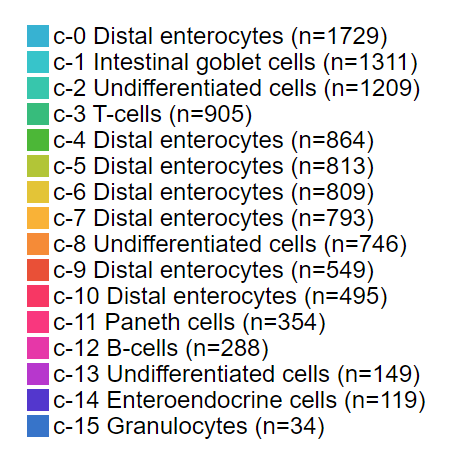
**

**A**

**
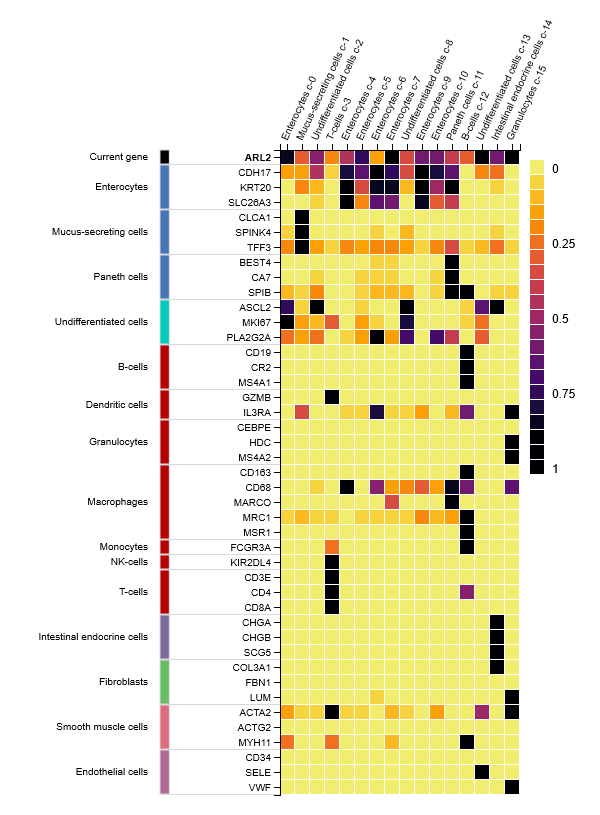
**

**B**

**Table S1.** List of antibodies and reagents used in this study

| **Antibodies** | | |
| --- | --- | --- |
| Target protein | Source | Identifier |
| ARL2 | Abcam (Cambridge, UK) | ab183510 |
| Cyclin A | Cell signaling (Danvers, MA) | #4656 |
| Cyclin B | Cell signaling | #4138 |
| Cyclin D | Cell signaling | #2978 |
| γ-H2AX | Cell signaling | #9718 |
| Histon H2A | Cell signaling | #2572 |
| Lamin A/C | Santa Cruz (Santa Cruz, CA) | SC-7292 |
| p53 | Santa Cruz | SC-126 |
| PARP1/2 | Santa Cruz | SC-7150 |
| GAPDH | Santa Cruz | SC-32233 |
| β-actin | Santa Cruz | SC-130657 |
| α-Tubulin | Santa Cruz | SC-23948 |
| Peroxidase AffiniPure Goat Anti-Mouse IgG (H+L) | Jackson ImmunoResearch (Philadelphia, PA) | 115-035-003 |
| Peroxidase AffiniPure Goat Anti-Rabbit IgG (H+L) | Jackson ImmunoResearch | 111-035-144 |
| **Reagents** | | |
| Name | Source | Identifier |
| Novex™ ECL Chemiluminescent Substrate Reagent Kit | Invitrogen (Carlsbad, CA) | WP20005 |
| SuperSignal™ West Femto Maximum Sensitivity Substrate | Thermo Fisher Scientific (Waltham, MA) | 34094 |
| WesternBright ECL HRP substrate | Advansta (San Jose, CA) | K-12045 |
| Lipofectamine™ RNAiMAX Transfection Reagent | Invitrogen | 13778075 |
| TransIT-X2® Dynamic Delivery System | Mirus Bio (Madison, WI) | MIR6000 |
| jetPRIME® transfection reagent | Polyplus (Illkirch, France) | 101000015 |
| Poly(2-hydroxyethyl methacrylate) | Sigma Aldrich (Burlington, MA) | P3932 |
| Hexadimethrine bromide | Sigma Aldrich | 107689 |
| Doxycycline hyclate | Sigma Aldrich | D9891 |
| T-PER™ Tissue Protein Extraction Reagent | Thermo Fisher Scientific | 78510 |
| cOmplete™ Protease Inhibitor Cocktail | Roche | 11697498001 |

**References S**

[1] Karlsson, M. et al. (2021). A single-cell type transcriptomics map of human tissues. Sci Adv 7

[2] Parikh, K. et al. (2019). Colonic epithelial cell diversity in health and inflammatory bowel disease. Nature 567, 49-55.
